# Tracheotomy

**Published:** 1890-04-19

**Authors:** 


### SURGERY.

### TRACHEOTOMY.

The *New England Medical* monthly journal recently contained an article by Dr. T. Marley, surgeon, Harlem Hospital, headed, "Traumatic Laryngitis," with a report of two cases, illustrating the difficulties attending the operation of tracheotomy. A great danger is congestion, swelling, and infiltration in the vicinity of the traumatism, and the first patient whose case is detailed died suddenly from œdema glottidis. In the second case, as the steel entered the rings of the trachea, blood came out in gushes, so that the operator, "had he not felt sure of his anatomy," would have thought he had punctured some large vessel. The difficulty was now to find the opening in the wind-pipe, it lying nearly an inch and a-half from the surface, in constant motion, while the blood swelled out in quantities. Blood also rushed into the trachea. After a tube was inserted little blood oozed out, and none entered the bronchi. Dr. Marley in conclusion observes that when an experienced operator cannot be had, and an operation in the air passages is imperative, it is well to simply pass a trochar and cannula into the crico-thyroid space instead of cutting. This requires no special skill, there will be no loss of blood, and it will relieve the engorged lungs. If anyone will simply measure the limited space which lies between the inferior border of the cricoid cartilage, the boundary of the larynx, and the upper border of the sternal notch, he will be surprised at the narrow limit of territory to which he is confined in performing tracheotomy. Now we perfectly well know that tracheotomy is sometimes a difficult operation, this depending much on the condition and physical features of the individual operated upon. But we doubt whether it would be advisable to perform laryngotomy instead, in cases of diphtheria for instance, when the parts below the larynx are often involved in the disease.

